# Long-term phenobarbital treatment is effective in working-age patients with epilepsy in rural Northeast China: a 10-year follow-up study

**DOI:** 10.3389/fneur.2024.1429964

**Published:** 2024-10-23

**Authors:** Rongxin Li, Danyang Zhao, Nan Li, Weihong Lin

**Affiliations:** Department of Neurology, The First Hospital of Jilin University, Changchun, China

**Keywords:** adult epilepsy, working-age population, phenobarbital efficacy, prognostic predictors, rural healthcare, adherence

## Abstract

**Introduction:**

Effective management of epilepsy in working-age patients is essential to reduce the burden on individuals, families, and communities. This study aimed to assess the long-term efficacy of phenobarbital (PB) in working-age patients with epilepsy in rural Northeast China and identify the risk factors for seizures during treatment.

**Methods:**

Patients aged 18–65 years diagnosed with convulsive epilepsy in rural areas of Jilin Province between 2010 and 2024 were included, and demographic and clinical data were recorded. Seizure frequency, self-efficacy, adherence, and adverse events (AEs) were assessed monthly.

**Results:**

Of the 3,568 participants, 288 (8.1%) withdrew from the study and 159 (4.5%) died. During the first year of treatment, 75.2% of patients experienced a ≥50% reduction in seizure frequency compared with baseline (considered as treatment effectiveness); 53.7% of patients were seizure-free. By the tenth year, 97.7% of patients showed treatment effectiveness, and 89.6% were seizure-free. Self-efficacy was improved in 37.8% of patients in the first year and in 72% of patients by the tenth year. The independent risk factors for seizures during treatment were higher baseline seizure frequency [odds ratio (OR) = 1.431, 95% confidence interval (CI): 1.122–1.824], presence of multiple seizure types (OR = 1.367, 95% CI: 1.023–1.826), and poor adherence (OR = 14.806, 95% CI: 3.495–62.725), with significant differences observed in the first, third, and fifth years. The most commonly reported AEs were drowsiness (43.3%), dizziness (25.0%), and headaches (17.0%), most of which were mild and decreased over time. Age at enrollment was the only factor influencing withdrawal (hazard ratio = 0.984, 95% CI: 0.973–0.996, *p* = 0.010), with a substantial number of patients who withdrew (32.6%) relocating for work. Cardiovascular disease was the primary cause of death, and age at enrollment was the only risk factor (hazard ratio = 1.026, 95% CI: 1.009–1.043, *p* = 0.002).

**Discussion:**

Working-age adults with epilepsy demonstrated a favorable response and tolerability to PB monotherapy. Baseline seizure frequency, seizure type, and adherence consistently predicted prognosis throughout the treatment period. Withdrawal was mainly explained by work-related pressures in this age group. Therefore, it is essential to implement interventions that support patient adherence to therapy and maintain stable regimens.

## 1 Introduction

Epilepsy, characterized by recurrent, unprovoked seizures (1), is a common chronic neurological disorder that affects over 50 million people worldwide (2). Given the considerable global health burden and economic costs of at least $110 billion (3), there is a need to provide effective treatments for adult patients with epilepsy, particularly those of working age (18–65 years) (4).

The prevalence of epilepsy increases in early adulthood and then stabilizes (5, 6). Early adulthood therefore represents a critical phase, with the interplay of genetic and environmental factors and neurobiological changes affecting the onset and progression of epilepsy. Disturbances in neuronal proliferation or migration during cerebral cortex development may lead to chronic epilepsy (7). In contrast, individuals lacking genetic factors typically experience seizures secondary to encephalitis/meningitis, traumatic brain injuries, or brain tumors (8). Neurobiological alterations are closely associated with age progression. Oxidative and nitrosative stresses (9), along with hormones, such as corticosteroids, estrogen, and progesterone, play crucial roles in modulating neuronal excitability and seizure susceptibility (10, 11). Adults are likely to be economically active and may be caregivers, and therefore are more severely affected by epilepsy than other age groups. Effects of epilepsy include stigma, lower educational attainment, and fewer work opportunities, resulting in loss of income and an inability to afford medical care (4, 12). Low marriage and fertility rates also compromise the quality of life in patients with epilepsy (13). Epilepsy therefore has significant detrimental effects on patients, as well as on their families and communities.

There is a dramatic global disparity in epilepsy treatment. More than 80% of individuals with epilepsy reside in middle- and low-income countries (14); however, these account for only 4% of the total global spending on epilepsy (3). In middle-income countries, the proportion of patients with active epilepsy who do not receive appropriate treatment can reach 50%, whereas in low-income countries this figure exceeds 75%. Treatment disparity also exists within countries, with access to appropriate care restricted in rural areas compared with suburban and urban settings (15). Epidemiological surveys conducted in rural China have shown that 63% of patients with active epilepsy did not receive treatment with the appropriate antiseizure medication (ASM) (16). The incidence of active epilepsy was also higher in rural areas than in the eastern coastal regions (4.6/1,000 compared with 2.4/1,000), which benefit from urbanization and improved economic conditions (17).

The Global Campaign Against Epilepsy was initiated in 1997 to address this gap in epilepsy treatment. In 2000, it was extended to rural areas of China, providing phenobarbital (PB) as a cost-free, potent, and reliable treatment option in these areas (16), and facilitating the study of long-term disease control. As over 40% of the population of Northeast China lives in rural areas, it is crucial to eliminate treatment disparities, particularly with regards to economically active adult patients. This prospective cohort study therefore aimed to assess PB treatment efficacy and adverse events (AEs), and identify risk factors for seizures during treatment, in working-age patients over a decade-long follow-up period.

## 2 Materials and methods

### 2.1 Participants

**Figure 1:**
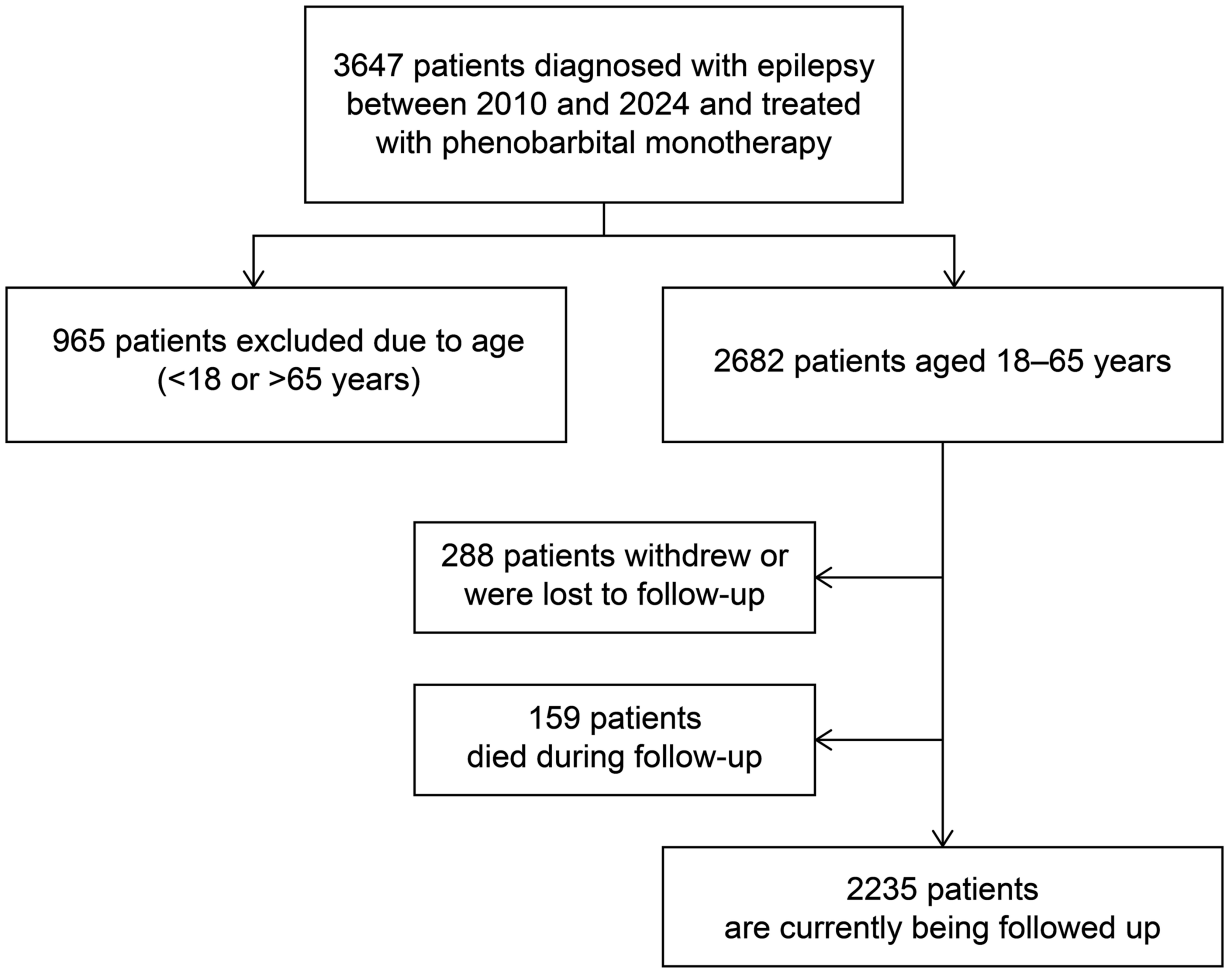
Flowchart of the study.

This prospective study recruited newly diagnosed patients with convulsive epilepsy aged 18–65 years from seven counties in Jilin Province, Northeast China between January 2010 and March 2024 (Figure 1). A diagnosis of convulsive epilepsy was made when at least two of the following symptoms were present: loss of consciousness, rigidity, and generalized convulsive movements, alongside any one of the following symptoms: urinary incontinence, bitten tongue, injury sustained in a fall, post-seizure fatigue, or post-seizure headache/muscle aches (16).

In addition to being 18–65 years of age, patients enrolled in the study met the following criteria: had no prior diagnosis of epilepsy, had experienced at least two unprovoked seizures more than 24 h apart within 1 year before enrollment, and had either none or irregular previous PB treatment. The exclusion criteria were as follows: seizures associated solely with pregnancy, alcohol or drug withdrawal; PB allergy; progressive neurological disease; history of status epilepticus; and current effective treatment with ASM.

Following enrollment, the diagnosis of epilepsy was confirmed by a neurologist and PB monotherapy was initiated; this therapy continued throughout the study period. All participants provided written informed consent and the study protocol was approved by the Ethics Committee of the First Hospital of Jilin University.

### 2.2 Follow-up and data collection

Participants were followed up through monthly door-to-door surveys or telephone interviews. During the coronavirus disease pandemic, data were collected via telephone to mitigate the risk of infection. Trained primary care physicians collected demographic and clinical data, including information on age, sex, seizure frequency, seizure type, prescribed drug dosage, and AEs. AEs included drowsiness, ataxia, dizziness, headaches, hyperactivity, skin rashes, gastrointestinal complaints, and anxiety/depression (18). Follow-ups also recorded adherence and self-reported physical, mental, and work/learning abilities (i.e., self-efficacy). A detailed description of the assessment methods for these measures are provided in the Supplementary material. Seizure-free status was defined as the absence of seizures for more than 12 consecutive months, and baseline seizure frequency was calculated from the number of seizures in the year before enrollment. Effective PB treatment was defined as achieving seizure-free status or a ≥50% reduction in seizure frequency compared with baseline. Conversely, ineffective PB treatment was defined as either an increase in seizure frequency compared with baseline or a <50% reduction in seizure frequency (19). If death occurred, the cause was ascertained via death certificates or by interviewing family members or neighbors of the deceased participant (20). Further classification of the cause of death was performed using the 10th Edition of the International Classification of Diseases.

### 2.3 Statistical analysis

Statistical analyses were conducted using SPSS software version 24.0 (IBM Corp., Armonk, NY, United States). The normality of continuous variables was assessed using the Shapiro–Wilk test. Non-normally distributed continuous data were presented as medians and ranges or interquartile ranges, whereas categorical data were presented as numbers and percentages and were compared using chi-squared or Fisher’s exact tests. The association between clinical characteristics and seizure remission was analyzed using the Mann–Whitney *U* test for skewed distribution and rank variables. Significant variables were included in a multivariate logistic regression analysis to identify factors associated with the risk of seizures at different follow-up time points. Cox regression models were used to identify risk factors for withdrawal from the study and death. Tests were two-tailed and significance was set at *p* < 0.05.

## 3 Results

### 3.1 Patient characteristics

**Figure 2:**
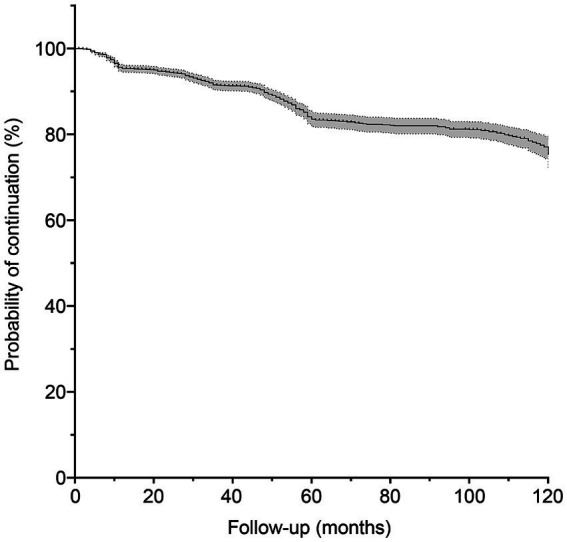
Probability of phenobarbital treatment continuation. The gray shaded area represents the 95% confidence interval.

This prospective study included 2,682 eligible patients enrolled between January 2010 and March 2024, of whom 288 withdrew from the study and 159 died during the follow-up period. The demographic and clinical characteristics of the patients are summarized in Table 1. The median age at enrollment was 43 years, with a median follow-up duration of 56 (range, 1–149) months. Seizure frequency was ≤6 in almost half of patients (46.9%, *n* = 1,391), and the majority of patients (84.9%, *n* = 2,515) experienced generalized tonic-clonic seizures. Figure 2 shows the estimated probability of PB monotherapy continuation over 10 years, which was 95.4, 91.5, 83.5, and 75.3% in the first, third, fifth, and tenth years, respectively.

**Table 1:** Demographic characteristics of patients with convulsive epilepsy.

Characteristics	Total	Patients who withdrew	Patients who died
Number	2,682	288	159
Sex			
Male	1,506 (56.2)	149 (51.7)	95 (59.7)
Female	1,176 (43.8)	139 (48.3)	64 (40.3)
Baseline age, years	43 (18–65)	41 (18–65)	46 (18–64)
Baseline BMI, kg/m^2^	23.0 (12.0–58.0)	22.9 (16.0–57.5)	23.1 (16.2–38.3)
Onset age, years	20 (0–65)	18 (0–65)	22 (0–60)
Disease duration, months	21 (1–65)	19 (1–55)	23 (1–59)
Baseline seizure frequency per year	8 (0–900)	6 (0–312)	7 (0–700)
≤6	1,260 (47.0)	150 (52.1)	74 (46.5)
7–12	541 (20.2)	57 (19.8)	35 (22.0)
13–36	563 (21.0)	56 (19.4)	27 (17.0)
>36	318 (11.9)	25 (8.7)	23 (14.5)
Baseline seizure type			
Generalized tonic-clonic	2,251 (83.9)	239 (83.0)	142 (89.3)
Focal to bilateral tonic-clonic	68 (2.5)	16 (5.6)	1 (0.6)
Other	231 (8.6)	10 (3.5)	12 (7.5)
Unclassified	132 (4.9)	23 (8.0)	4 (2.5)
Follow-up period, months	58 (1–149)	47 (1–149)	34 (4–120)

Data are expressed as *n* (%) or median (range). BMI, body mass index.

### 3.2 Efficacy of PB treatment

From the study cohort, 2,235 patients were followed up. Currently, 81.7% (*n* = 1,825) of these patients have achieved a seizure-free status, 10.2% (*n* = 229) experienced at least a 50% reduction in seizure frequency, and 8.1% (*n* = 181) exhibited a <50% reduction. As the patients were enrolled at different times, an analysis of data from the first, third, fifth, and tenth years of follow-up was conducted to assess the long-term efficacy of PB treatment. Table 2 provides an overview of the changes in seizure frequency during the first (*n* = 2,358), third (*n* = 2,115), fifth (*n* = 1,671), and tenth (*n* = 665) years of PB monotherapy. In the first year, 75.2% of patients (*n* = 1,772) experienced at least a 50% reduction in seizure frequency, with 53.7% (*n* = 1,266) achieving seizure-free status. The treatment effectiveness rate increased over time, reaching 97.7% (*n* = 650) in the tenth year; only 2.3% of patients (*n* = 15) showed a <50% reduction in seizure frequency.

**Table 2:** PB monotherapy effects, dosage, and adverse events in patients with epilepsy.

	First year	Third year	Fifth year	Tenth year
	(*n* = 2,358)	(*n* = 2,115)	(*n* = 1,671)	(*n* = 665)
Seizure-free	1,266 (53.7)	1,590 (75.2)	1,444 (86.4)	596 (89.6)
≥50% reduction in seizure frequency (but not seizure-free)	506 (21.5)	307 (14.5)	164 (9.8)	54 (8.1)
<50% reduction in seizure frequency	586 (24.9)	218 (10.3)	63 (3.8)	15 (2.3)
Dosage of PB (mg/day)	60 (60–90)	90 (60–120)	90 (60–120)	90 (60–120)
Adverse events	1,167 (49.5)	862 (40.8)	602 (36.0)	178 (26.8)
Drowsiness	1,022 (43.3)	749 (35.4)	520 (31.1)	153 (23.0)
Ataxia	311 (13.2)	226 (10.7)	122 (7.3)	16 (2.4)
Dizziness	589 (25.0)	435 (20.6)	298 (17.8)	59 (8.9)
Headaches	401 (17.0)	258 (12.2)	168 (10.1)	33 (5.0)
Hyperactivity	173 (7.3)	108 (5.1)	69 (4.1)	0 (0)
Skin rashes	114 (4.8)	68 (3.2)	19 (1.1)	3 (0.5)
Gastrointestinal complaints	199 (8.4)	96 (4.5)	67 (4.0)	10 (1.5)
Anxiety or depression	168 (7.1)	121 (5.7)	76 (4.5)	0 (0)

Data are expressed as *n* (%) or median (interquartile range). PB, phenobarbital.

A similar trend was observed in patient-reported self-efficacy assessments (Table 3). The majority of patients (55.2%, *n* = 1,086) reported no enhancement in their physical, mental, or work/learning abilities during the first year, with only 37.8% of patients (*n* = 744) perceiving an improvement. This percentage was increased over time, and by the tenth year, 72% of patients (*n* = 167) perceived a positive change.

**Table 3:** Changes in self-efficacy during phenobarbital monotherapy.

	First year	Third year	Fifth year	Tenth year
	(*n* = 1,967)	(*n* = 1,751)	(*n* = 1,584)	(*n* = 292)
Improvement	744 (37.8)	754 (43.1)	805 (50.8)	167 (72.0)
No change	1,086 (55.2)	933 (53.3)	751 (47.4)	54 (23.3)
Deterioration	137 (7.0)	64 (3.7)	28 (1.8)	11 (4.7)

Data are expressed as *n* (%).

**Figure 3:**
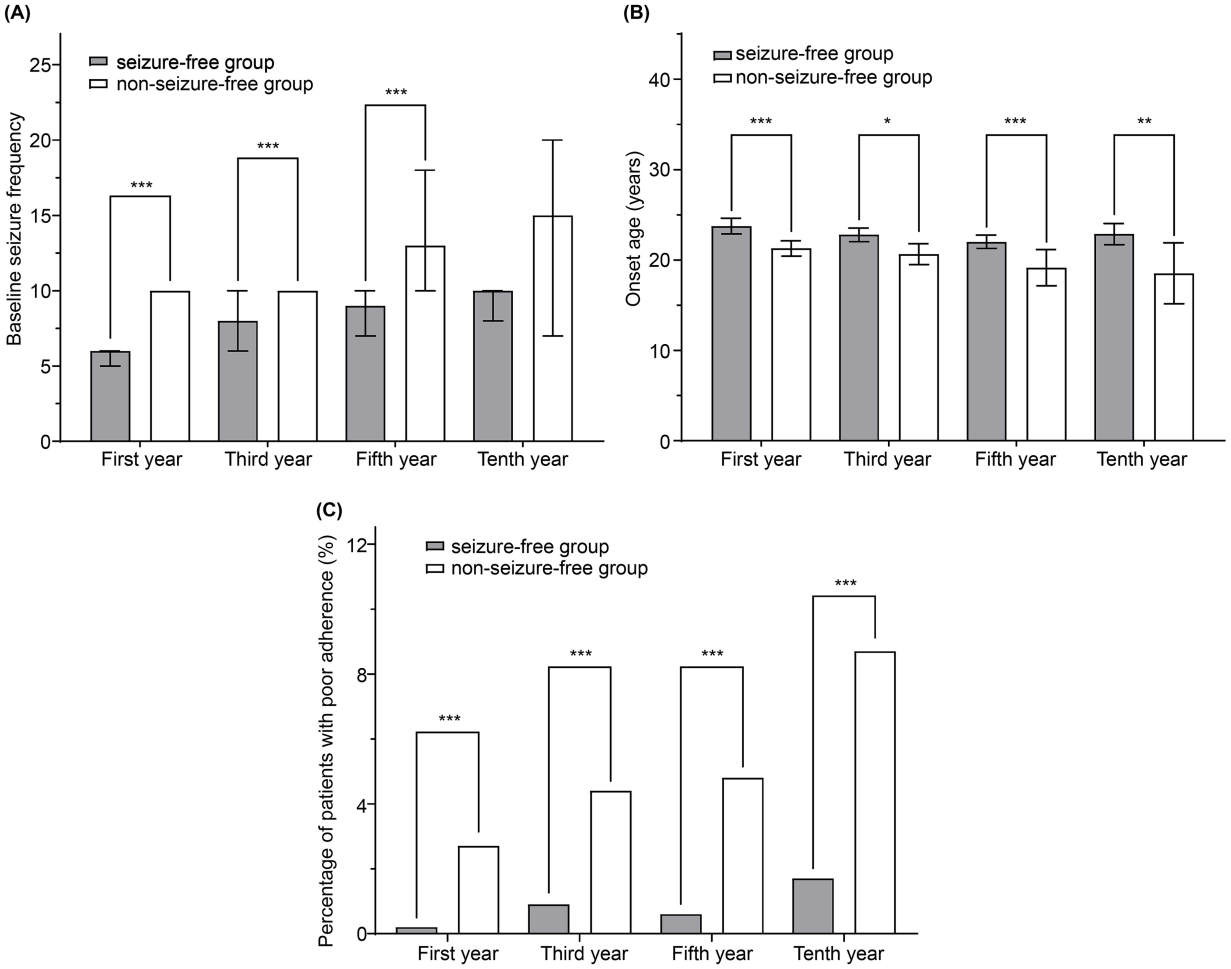
Comparison of seizure-free and non-seizure-free groups during follow-up. **(A)** Baseline seizure frequency, **(B)** age at onset, and **(C)** percentage of patients with poor adherence were compared between the two groups. The bars and lines represent the mean ± 95% confidence interval. ^*^p < 0.05, ^**^p < 0.01, and ^***^p < 0.001.

Figure 3 compares the seizure-free and non-seizure-free groups at the first, third, fifth, and tenth years of follow-up. Results of the statistical analyses of the differences between these groups are summarized in Supplementary Table S1. Significant differences in age at onset, disease duration before enrollment, baseline seizure frequency, baseline seizure type, level of consciousness at seizure onset, and adherence were observed between the groups during the first year of follow-up (*p* < 0.05). In the third and fifth years, significant differences persisted in the age at onset, disease duration, baseline seizure type, baseline seizure frequency, and adherence. Significant differences between the two groups in age at onset and adherence persisted in the tenth year. Physicians classified adherence levels of patients as good or poor. Patients with good adherence were more likely to be seizure-free at all time points, including the first year.

Variables that differed significantly between the seizure-free and non-seizure-free groups (*p* < 0.05) were included in a multivariate logistic regression analysis to identify independent risk factors for seizures (Table 4). Owing to insufficient patient numbers in the tenth year, the analysis was performed only on data from the first, third, and fifth years of follow-up. Logistic regression analysis revealed that higher baseline seizure frequency [odds ratio (OR) = 1.431, 95% confidence interval (CI): 1.122–1.824 for the 7–12 seizures subgroup; OR = 2.092, 95% CI: 1.643–2.665 for the 13–36 seizures subgroup; and OR = 2.354, 95% CI: 1.762–3.144 for the >36 seizures subgroup], multiple seizure types (OR = 1.367, 95% CI: 1.023–1.826), and poor adherence (OR = 14.806, 95% CI: 3.495–62.725) were associated with an increased risk of seizures in the first year. These variables remained independent risk factors for seizures during the third and fifth years of treatment, although the effect of baseline seizure frequency on seizure risk was limited to patients with higher seizure frequencies at the later time points.

**Table 4:** Multivariate logistic regression analysis to identify factors predictive of seizures.

	First year			Third year			Fifth year		
	*B*	OR (95% CI)	*p*-value	*B*	OR (95% CI)	*p*-value	*B*	OR (95% CI)	*p*-value
Baseline age, years				−0.009	0.991 (0.980–1.002)	0.112			
Onset age, years	−0.003	0.997 (0.989–1.005)	0.451	−0.003	0.997 (0.988–1.007)	0.587	−0.007	0.993 (0.979–1.008)	0.370
Disease duration, months	0.004	1.004 (0.995–1.013)	0.412				0.004	1.004 (0.989–1.019)	0.619
Baseline seizure frequency per year									
≤6 (ref)			<0.001			<0.001			<0.001
7–12	0.358	1.431 (1.122–1.824)	0.004	0.129	1.138 (0.836–1.549)	0.410	−0.045	0.956 (0.607–1.504)	0.845
13–36	0.738	2.092 (1.643–2.665)	<0.001	0.445	1.560 (1.171–2.079)	0.002	0.698	2.009 (1.378–2.930)	<0.001
>36	0.856	2.354 (1.762–3.144)	<0.001	0.647	1.909 (1.379–2.642)	<0.001	0.542	1.719 (1.101–2.686)	0.017
Baseline seizure type	0.313	1.367 (1.023–1.826)	0.034	0.358	1.431 (1.001–2.045)	0.049	0.766	2.151 (1.248–3.708)	0.006
Unconsciousness at seizure	0.169	1.184 (0.802–1.748)	0.396	0.370	1.448 (0.910–2.303)	0.118			
Adherence	2.695	14.806 (3.495–62.725)	<0.001	1.697	5.456 (2.757–10.797)	<0.001	2.137	8.473 (3.341–21.486)	<0.001

CI, confidence interval; OR, odds ratio.

### 3.3 AEs

We also evaluated the incidence of AEs in working-age patients receiving PB monotherapy. The most common AEs in the first year were drowsiness (43.3%), dizziness (25.0%), headaches (17.0%), and ataxia (13.2%); skin rashes were the least common AE (4.8%). Most AEs were mild. Detailed information on all AEs is provided in Table 2. The distribution of AEs remained similar at the later time points, although the overall incidence of AEs decreased over time. Supplementary Table S2 examines the impact of AEs on adherence. In the first year of follow-up, patients in the group with AEs were significantly more likely to have poor adherence, compared with those without AEs (*p* = 0.013). This impact diminished over time, because the difference was no longer significant in later follow-ups.

### 3.4 Withdrawal

During the follow-up period, 288 patients withdrew from the treatment program and study. The main reasons for withdrawal were nonadherence (43.1%, *n* = 124, including those who discontinued PB treatment due to unsatisfactory efficacy) and relocation outside the study area (32.6%, *n* = 94). Missed visits (11.8%, *n* = 34), AEs (8.0%, *n* = 23), and pregnancy or other systemic diseases (4.5%, *n* = 13) also led to withdrawals. The hazard ratio for withdrawal was analyzed using Cox regression analysis, adjusting for demographic and clinical characteristics, including sex, age at enrollment, age at onset, baseline seizure frequency, baseline seizure type, and level of consciousness at seizure onset. Additionally, AEs considered potential risk factors for withdrawal were taken into account. The results indicated a significant decrease in the risk of withdrawal with increasing patient age at enrollment (hazard ratio = 0.984, 95% CI: 0.973–0.996, *p* = 0.010).

### 3.5 Death

A total of 159 patients died during the study, at a median of 34 (interquartile range, 10–56) months. As shown in Table 5, the leading cause of death was cardiac disease (36.5%, *n* = 58), followed by cerebrovascular disease (30.8%, *n* = 49), other systemic disease (15.1%, *n* = 24), cancer (9.4%, *n* = 15), accident (3.8%, *n* = 6), status epilepticus (2.5%, *n* = 4), and unknown cause (1.9%, *n* = 3). The Cox proportional hazards model, adjusted for sex, age at enrollment, age at onset, baseline seizure frequency, baseline seizure type, level of consciousness at seizure onset, and AEs, revealed that an increased age at enrollment was associated with a higher risk of death (hazard ratio = 1.026, 95% CI: 1.009–1.043, *p* = 0.002).

**Table 5:** Causes of death in patients with epilepsy during the study period.

Cause	*n* (total, *n* = 159)	%
Cardiac disease	58	36.5
Cerebrovascular disease	49	30.8
Hemorrhagic stroke	19	11.9
Ischemic stroke	30	18.9
Other systemic disease	24	15.1
Cancer	15	9.4
Accident	6	3.8
Status epilepticus	4	2.5
Unknown	3	1.9

## 4 Discussion

Epilepsy poses a significant global public health challenge, affecting people of all ages and backgrounds. Although various ASMs are available, the cost-effective ASM PB remains widely used, especially in areas where resources are scarce and access to novel ASMs may be limited (18).

Seizures may arise from congenital defects in neural networks or from acquired brain structural abnormalities, infections, or metabolic issues (21). Consequently, patients of different ages exhibit distinct etiological characteristics. In adults, the impact of genetic and developmental factors diminishes gradually with age, whereas the influence of cumulative brain damage increases. Studies have indicated that the incidence of active convulsive epilepsy peaks at the age of 30 to 39 years (22). However, individuals in this age group often experience delayed diagnosis and treatment, compared with pediatric or older patients (23). It has been demonstrated that over 80% of individuals diagnosed with epilepsy require treatment with ASMs, with 65–70% achieving seizure control with standard ASMs (24). Economically active adult patients face concerns related to labor force exclusion and low productivity as well as medical issues (22, 25, 26). Therefore, improved treatment of these patients is crucial, especially in low-income areas, as effective epilepsy control can significantly reduce the health and economic burden on families and communities (3).

According to a previous study, approximately 50% of patients with newly diagnosed epilepsy achieve complete seizure control using ASMs (27). PB is an ASM with initial efficacy similar to those of phenytoin, carbamazepine, levetiracetam, and lamotrigine (28–30), and therefore represents a cost-effective option for epilepsy treatment in developing countries (31). In Mali, up to 80.2% of patients treated with PB were seizure-free within 5 months (32). A study conducted in Nigeria reported that 50.6% of the patients treated with PB achieved seizure-free status (33). Similarly, studies in China reported that 39–46% of patients were seizure-free after 1 year of PB treatment, which had an overall treatment efficacy of 68–77% (34–36). Our study found that 53.7% of patients achieved seizure-free status, with an overall treatment efficacy of 75.2% in the first year. These results confirm those of previous studies demonstrating the efficacy of PB in early seizure management. The rapid response to PB results in a prompt reduction in seizure frequency, instilling confidence in patients and allowing them to swiftly return to normal daily life and productivity, thereby alleviating the burden of epilepsy on individuals and communities.

Our finding showing that the effectiveness of PB treatment gradually improved over time is consistent with the results of several previous reports of community-based management programs in rural China. A 2-year trial of PB monotherapy found that over 70% of patients showed continuous significant improvement (16). Extending the follow-up period to 6 years revealed that most patients (88%) remained seizure-free, indicating that PB therapy has sustained benefits (34). A decade-long study of female patients with epilepsy reported a seizure-free rate of 96.6% in the tenth year; however, this result should be interpreted with caution owing to the small sample size of 59 patients (36). The present study revealed a similar seizure-free rate of 89.6% based on a much larger sample size of 665 patients in the tenth year. These findings confirm that PB exerts rapid and enduring effects in most patients. PB is therefore a reliable therapeutic option for low-income patients residing in rural areas.

Multivariate logistic regression analysis confirmed that baseline seizure frequency, seizure type, and adherence were independent risk factors for seizure occurrence during long-term treatment. Specifically, baseline seizure frequency is widely regarded as a reliable prognostic indicator, with higher frequencies leading to increased risk. A British study showed that baseline seizure frequency was inversely correlated with the remission rate (37). Similarly, a retrospective cohort study in Ethiopia demonstrated that patients with a higher baseline seizure burden were 36% less likely to achieve seizure remission than those with a lower baseline seizure burden (38). Our study further supports the significant impact of baseline seizure frequency on long-term outcomes, particularly in high-frequency cases. Frequent seizures indicates severe disease and may decrease the efficacy of pharmacological treatment (39). In addition, prolonged or recurrent epileptic seizures may lead to neuronal damage and the subsequent formation of new epileptic foci (40). Therefore, baseline seizure frequency provides clinicians with crucial insights into disease prognosis and aids in the tailoring of individualized treatment plans.

The influence of baseline seizure type on the risk of seizures persisted throughout the follow-up period, with multiple seizure types being associated with poor treatment outcomes and increased seizure risk. The co-occurrence of seizure types has previously been shown to be a risk factor for drug resistance (41), significantly increasing the probability of recurrent generalized tonic-clonic seizures (42). Plausible hypotheses include aberrant functional connectivity, minor focal lesions, and sustained interneuron damage (43). The simultaneous presence of different seizure types induces cortical coactivation and therefore widespread changes in the brain network, contributing to poor outcomes.

Adherence is crucial for controlling epilepsy, and it has previously been estimated that 31% of seizures could be attributed to nonadherence (44). Our study confirmed that adherence significantly influenced the risk of seizures during the follow-up period, demonstrating that patients with poor adherence experienced a marked increase in seizure frequency. During the first year of therapy, patients with poor adherence had a 14.8 times higher risk of seizures, compared with those with good adherence. An approach that focuses on ASM selection and dosage may have limited effectiveness if no attempt is made to encourage adherence. Indeed, the World Health Organization asserts that good adherence may impact population health more than improving specific medical therapies (45). Enhancing adherence has been shown to improve seizure control by approximately 64% (46–48) and ultimately lead to better patient outcomes.

Similar to other ASMs, PB may induce specific dose-dependent adverse reactions (18). Clinical trials conducted in developed countries have described a wide range of central nervous system AEs associated with PB, including drowsiness, behavioral issues, cognitive impairment, and depressive symptoms (49). In contrast, several observational studies conducted in developing countries have reported high levels of efficacy and tolerability with no associated toxicity burden or cognitive decline (50, 51), and our study observed that AEs related to PB therapy were typically mild and tended to diminish over time. This variability may arise from differences in the prescribed dosages. The unique mechanism of action of gamma-aminobutyric acid is thought to enable PB to achieve high efficacy even at lower doses (52). For economically active adults, it is particularly important that treatments are tolerable, allowing a return to normal life, and the favorable tolerability of PB therefore recommends it for clinical practice.

Our study confirms that AEs affect adherence, although not in a linear manner. Early in treatment, compared with patients without AEs, those with AEs are more likely to exhibit poor adherence, even though most AEs are mild. This is primarily due to the increased sensitivity of patients to AEs when they first start taking PB, which can sometimes outweigh the perceived need for the medicine (44). Concerns about potential toxicity, possibly due to heightened anxiety, may also lead to discontinuation of PB. Over time, patients typically develop tolerance to PB, and their belief in the treatment and trust in physicians often increase, which mitigates the impact of AEs on adherence. Our study found that nearly half of the patients who withdrew from the treatment program and study did so because of nonadherence, whereas only a minority cited severe AEs. There was a higher likelihood of treatment discontinuation among younger patients, which we hypothesized may have stemmed from a lack of understanding of the importance of standardized therapy rather than an increased rate of AEs. Additionally, patients withdrew from our study because of relocation, in higher numbers than those reported previously in studies of other age groups (16, 35). This is possibly because working-age adults are more likely to move for educational and work opportunities. To address these challenges, implementing comprehensive multidimensional strategies is essential. Physicians should focus on AEs during early treatment, providing ample support and education to help patients adapt and minimize the negative impact of AEs on adherence. In long-term treatment, managing multiple factors affecting adherence is crucial, including psychological support, disease education, socioeconomic assistance, and healthcare accessibility (53). Regular evaluation and adjustment of treatment plans are essential to ensure that all factors influencing adherence are effectively addressed.

Patients with epilepsy are two to three times more likely to die prematurely than the general population (54, 55). Cox regression analysis revealed that the risk of death was increased with age at baseline, which was consistent with the results of previous studies (35, 36). In our study, heart disease and stroke were the primary causes of mortality. Stroke can induce adult-onset epilepsy, whereas seizures may precipitate hypertension, cerebral small vessel disease, and cerebral hemorrhage (56). The interplay between epilepsy and cardiovascular disease is complex. Recurrent seizures may trigger catecholamine release and hypoxemia, leading to changes in the myocardial and coronary vasculature (57). Working-age individuals are more likely to die from cardiovascular diseases than older patients with epilepsy (35). This may be attributed to the less apparent subclinical cerebrovascular lesions compared with the mechanical and electrical dysfunctions of the heart at this age, although further research is needed to confirm this hypothesis. Nevertheless, early seizure control can benefit patients by reducing chronic cardiovascular and cerebrovascular damage.

Our study, which spanned 10 years and enrolled a substantial patient cohort, allowed a comprehensive longitudinal analysis of PB therapy, yielding reliable results. By recruiting economically active participants, we have studied the ability of a cost-effective therapy to alleviate the burden on individuals, families, and society. This perspective distinguishes our study from previous reports. However, this study has certain limitations. Data collection through door-to-door or telephone interviews may have compromised the data quality. To ensure the reliability of the results, we did not impute or modify any missing data in the original dataset. Moreover, the scarcity of medical resources and diagnostic tools in rural areas led to shortages of blood tests, neuroimaging examinations, and electroencephalograms. Owing to the lower income levels in rural areas and limited access to ASMs in primary care, many patients, despite poor seizure control, continued free PB monotherapy without transitioning to more standardized epilepsy treatments. Future studies should aim to improve the accessibility of ASMs in rural regions and document detailed treatment changes, including medication switches and add-ons, to more comprehensively assess treatment efficacy and the impact on AEs. Additionally, although our study included the period of the coronavirus disease pandemic, it did not specifically investigate its effect on the risk of seizures.

## 5 Conclusion

PB monotherapy is effective in working-age patients with epilepsy over a long period. The independent risk factors for seizures during treatment include high baseline seizure frequency, multiple seizure types, and poor adherence. Therefore, improving adherence may represent a cost-effective method of optimizing treatment efficacy. PB therapy was generally well tolerated, with most AEs being mild. The increased likelihood of younger patients relocating for educational and work opportunities, and their increased risk of cardiovascular disease, also needs to be considered when treating this population. This study provides insights into the efficacy of PB monotherapy, and the risk factors of seizures during treatment, within a specific age group over an extended follow-up period. The future implementation of multifaceted interventions is crucial to enhance adherence, achieve early seizure control, and facilitate the return to work.

## Data Availability

The original contributions presented in the study are included in the article/Supplementary material, further inquiries can be directed to the corresponding author.
