# Supplementary material for: Long-term phenobarbital treatment is effective in working-age patients with epilepsy in rural Northeast China: a 10-year follow-up study
Source: Front Neurol. 2024 Oct 23;15:1429964. doi: 10.3389/fneur.2024.1429964 (PMC11538064; doi:10.3389/fneur.2024.1429964)
Supplement: Supplementary file 1 [file Data_Sheet_1.docx]

Supplementary Material

Long-term Phenobarbital Treatment is Effective in Working-age Patients with Epilepsy in Rural Northeast China: A 10-year Follow-up Study

**Supplementary Methods**

**Adherence Assessment**

Adherence was measured using two methods: (i) Appointment attendance, where patients were required to attend follow-up appointments every 2 weeks for the first 2 months and monthly after that, and (ii) Pill counts, where doses were monitored through counts of residual phenobarbital tablets. Patients were considered to have poor medication compliance if they exceeded or missed more than one daily dose within a 2-week interval or more than two daily doses within a 4-week interval. Poor adherence was identified if patients failed to attend a scheduled follow-up appointment without a valid excuse or demonstrated poor medication compliance for three consecutive periods.

**Self-Efficacy Assessment**

Self-efficacy was defined as the patient's perceived ability to manage physical, mental, and work/learning activities. It was assessed based on patients' self-reports during follow-ups. (i) Physical capability: The perceived ability to manage daily activities and physical health. (ii) Mental health: The perceived ability to handle emotional well-being and stress management. (iii) Work/learning capability: Confidence in managing work or academic responsibilities.

**Supplementary Table 1.** Comparison of baseline characteristics and adherence across different treatment outcomes

Data are expressed as n (%) or median (minimum–maximum). BMI, body mass index

|  | First year | | |  | Third year | | |  | Fifth year | | |  | Tenth year | | |  |
| --- | --- | --- | --- | --- | --- | --- | --- | --- | --- | --- | --- | --- | --- | --- | --- | --- |
|  | Seizure-free | Non-seizure-free | z | *p*-value | Seizure-free | Non-seizure-free | z | *p*-value | Seizure-free | Non-seizure-free | z | *p*-value | Seizure-free | Non-seizure-free | z | *p*-value |
|  | (n=1266) | (n=1092) |  |  | (n=1590) | (n=525) |  |  | (n=1444) | (n=227) |  |  | (n=596) | (n=69) |  |  |
| Sex |  |  | -1.626 | 0.104 |  |  | -1.029 | 0.303 |  |  | -0.245 | 0.807 |  |  | -0.558 | 0.577 |
| Male | 732 (57.8) | 595 (54.5) |  |  | 901 (56.7) | 284 (54.1) |  |  | 795 (55.1) | 123 (54.2) |  |  | 332 (55.7) | 36 (52.2) |  |  |
| Female | 534 (42.2) | 497 (45.5) |  |  | 689 (43.3) | 241 (45.9) |  |  | 649 (44.9) | 104 (45.8) |  |  | 264 (44.3) | 33 (47.8) |  |  |
| Baseline age, years | 44 (19–65) | 43 (18–65) | -1.771 | 0.077 | 43 (18–65) | 42 (18–65) | -2.759 | 0.006 | 42 (18–65) | 41 (19–65) | -0.890 | 0.373 | 43 (18–65) | 38 (19–62) | -2.064 | 0.039 |
| Baseline BMI, kg/m^2^ | 23.0 (13.0–58.0) | 22.9 (12.0–47.0) | -0.456 | 0.648 | 23.0 (13.3–57.5) | 22.9 (11.7–46.9) | -0.522 | 0.602 | 23.0 (11.7–57.5) | 23.0 (16.3–46.9) | -0.524 | 0.600 | 22.7 (11.7–41.5) | 23.4 (17.0–33.8) | -0.918 | 0.358 |
| Onset age, years | 20 (0–65) | 19 (0–65) | -3.599 | <0.001 | 20 (0–65) | 19 (0–63) | -2.249 | 0.025 | 20 (0–65) | 16 (0–60) | -3.497 | <0.001 | 20 (0–60) | 17 (1–60) | -2.560 | 0.010 |
| Disease duration, months | 20 (1–62) | 21 (1–66) | -3.550 | <0.001 | 21 (1–62) | 21 (1–66) | -1.129 | 0.259 | 20 (1–62) | 23 (1–53) | -2.340 | 0.019 | 20 (1–56) | 19 (1–57) | -0.639 | 0.523 |
| Baseline seizures frequency per year |  |  | -9.150 | <0.001 |  |  | -4.048 | <0.001 |  |  | -4.364 | <0.001 |  |  | -1.883 | 0.060 |
| ≤6 | 681 (53.8) | 417 (38.2) |  |  | 756 (47.5) | 212 (40.4) |  |  | 665 (46.1) | 76 (33.5) |  |  | 264 (44.3) | 25 (36.2) |  |  |
| 7–12 | 251 (19.8) | 221 (20.2) |  |  | 323 (20.3) | 97 (18.5) |  |  | 303 (21.0) | 37 (16.3) |  |  | 127 (21.3) | 8 (11.6) |  |  |
| 13–36 | 220 (17.4) | 279 (25.5) |  |  | 332 (20.9) | 125 (23.8) |  |  | 392 (20.2) | 72 (31.5) |  |  | 131 (22.0) | 23 (33.3) |  |  |
| >36 | 114 (9.0) | 175 (16.0) |  |  | 179 (11.3) | 91 (17.3) |  |  | 184 (12.7) | 42 (18.5) |  |  | 74 (12.4) | 13 (18.8) |  |  |
| Baseline seizure type |  |  | -2.403 | 0.016 |  |  | -2.034 | 0.042 |  |  | -2.489 | 0.013 |  |  | -1.624 | 0.104 |
| Generalized tonic-clonic | 1088 (85.9) | 897 (82.1) |  |  | 1388 (87.3) | 442 (84.2) |  |  | 1265 (87.6) | 213 (93.8) |  |  | 523 (87.8) | 65 (94.2) |  |  |
| Focal to bilateral tonic-clonic | 17 (1.3) | 35 (3.2) |  |  | 36 (2.3) | 15 (2.9) |  |  | 46 (3.2) | 4 (1.8) |  |  | 26 (4.4) | 2 (2.9) |  |  |
| Other types | 109 (8.6) | 104 (9.5) |  |  | 111 (7.0) | 25 (4.8) |  |  | 104 (7.2) | 9 (4.0) |  |  | 32 (5.4) | 2 (2.9) |  |  |
| Unclassified | 52 (4.1) | 56 (5.1) |  |  | 55 (3.5) | 43 (8.2) |  |  | 29 (2.0) | 1 (0.4) |  |  | 15 (2.5) | 0 (0) |  |  |
| Unconsciousness at seizure |  |  | -3.336 | <0.001 |  |  | -4.034 | <0.001 |  |  | -1.310 | 0.190 |  |  | -0.149 | 0.882 |
| None | 168 (13.3) | 127 (11.6) |  |  | 129 (8.1) | 74 (14.1) |  |  | 89 (6.2) | 9 (4.0) |  |  | 32 (5.4) | 4 (5.8) |  |  |
| Sometimes | 332 (26.2) | 227 (20.8) |  |  | 420 (26.4) | 93 (17.7) |  |  | 400 (27.7) | 64 (28.2) |  |  | 173 (29.0) | 23 (33.3) |  |  |
| Every time | 766 (60.5) | 738 (67.6) |  |  | 1041 (65.5) | 358 (68.2) |  |  | 955 (66.1) | 154 (67.8) |  |  | 391 (65.6) | 42 (60.9) |  |  |
| Adherence |  |  | -9.499 | <0.001 |  |  | -5.224 | <0.001 |  |  | -5.516 | <0.001 |  |  | -3.678 | <0.001 |
| Good adherence | 995 (78.6) | 941 (86.2) |  |  | 1295 (81.4) | 419 (79.8) |  |  | 1317 (91.2) | 193 (85.0) |  |  | 568 (95.3) | 59 (85.5) |  |  |
| Poor adherence | 2 (0.2) | 29 (2.7) |  |  | 14 (0.9) | 23 (4.4) |  |  | 9 (0.6) | 11 (4.8) |  |  | 10 (1.7) | 6 (8.7) |  |  |
| Unknown | 269 (21.2) | 122 (11.2) |  |  | 281 (17.7) | 83 (15.8) |  |  | 118 (8.2) | 23 (10.1) |  |  | 18 (3.0) | 4 (5.8) |  |  |

“Other types” represent a combination of two or more seizure types.

**Supplementary Table 2.** Comparison of adherence rates between patients with and without AEs

|  | First year | | |  | Third year | | |  | Fifth year | | |  | Tenth year | | |  |
| --- | --- | --- | --- | --- | --- | --- | --- | --- | --- | --- | --- | --- | --- | --- | --- | --- |
|  | AEs | Non-AEs | z | *p*-value | AEs | Non-AEs | z | *p*-value | AEs | Non-AEs | z | *p*-value | AEs | Non-AEs | z | *p*-value |
|  | (n=1167) | (n=1191) |  |  | (n=862) | (n=1253) |  |  | (n=602) | (n=1069) |  |  | (n=178) | (n=487) |  |  |
| Adherence |  |  | -2.480 | 0.013 |  |  | -1.510 | 0.131 |  |  | -1.173 | 0.241 |  |  | -0.770 | 0.441 |
| Good adherence | 1002 (85.9) | 934 (78.4) |  |  | 759 (88.1) | 955 (76.2) |  |  | 562 (93.4) | 948 (88.7) |  |  | 172 (96.6) | 455 (93.4) |  |  |
| Poor adherence | 23 (2.0) | 8 (0.7) |  |  | 21 (2.4) | 16 (1.3) |  |  | 10 (1.6) | 10 (0.9) |  |  | 3 (1.7) | 13 (2.7) |  |  |
| Unknown | 142 (12.2) | 249 (20.9) |  |  | 82 (9.5) | 282 (22.5) |  |  | 30 (5.0) | 111 (10.4) |  |  | 3 (1.7) | 19 (3.9) |  |  |

Data are expressed as n (%). AE, adverse events
